# Regional economic forecast using Elman neural networks with wavelet function

**DOI:** 10.1371/journal.pone.0299657

**Published:** 2024-03-07

**Authors:** Huade Liang, Huilin Zeng, Xiaojuan Dong

**Affiliations:** Guangzhou Nanyang Polytechnic College, Guangdong, China; National Institute of Technology, India (Institute of National Importance), INDIA

## Abstract

Recently, the economy in Guangdong province has ranked first in the country, maintaining a good growth momentum. The prediction of Gross Domestic Product (GDP) for Guangdong province is an important issue. Through predicting the GDP, it is possible to analyze whether the economy in Guangdong province can maintain high-quality growth. Hence, to accurately forecast the economy in Guangdong, this paper proposed an Elman neural network combining with wavelet function. The wavelet function not only stimulates the forecast ability of Elman neural network, but also improves the convergence speed of Elman neural network. Experimental results indicate that our model has good forecast ability of regional economy, and the forecast accuracy reach 0.971. In terms of forecast precision and errors, our model defeats the competitors. Moreover, our model gains advanced forecast results to both individual economic indicator and multiple economic indicators. This means that our model is independently of specific scenarios in regional economic forecast. We also find that the investment in education has a major positive impact on regional economic development in Guangdong province, and the both surges positive correlation. Experimental results also show that our model does not exhibit exponential training time with the augmenting of data volume. Consequently, we propose that our model is suitable for the prediction of large-scale datasets. Additionally, we demonstrate that using wavelet function gains more profits than using complex network architectures in forecast accuracy and training cost. Moreover, using wavelet function can simplify the designs of complexity network architectures, reducing the training parameter of neural networks.

## 1. Introduction

Guangdong province attaches great importance to regional coordinated development and vigorously implements the strategy of regional revitalization and development, therefore, the trend of regional economic disparities in the province has slowed down. However, there are shortcomings in infrastructure construction and equalization of basic public services. Consequently, it is an urgent task to narrow the economic gap between regions for economic development in Guangdong province.

With the high-quality development of regional economy, higher physical education in Guangdong province has achieved significant results. The development of higher physical education has become an important indicator affecting the economic development of Guangdong province. To provide suggestions for the economic development of Guangdong province, through analyzing various indicators, e.g., the development of higher physical education, the establishment of public fitness facilities, etc., neural network methods were applied to predict the economy of this province.

The historical data of regional economic development is extremely complex, including many influencing factors. However, due to complex relationships between data, it is difficult to find indicators that objectively and comprehensively reflect economic development. Traditional analysis methods, such as multiple linear regression [1, 2], grey correlation model [3], and time series [4], cannot effectively analyze indicators with high regional economic linearity, and cannot obtain accurately economic prediction. With the development of nonlinear dynamics technology, there are various predictive techniques with strong nonlinear analysis capabilities that can comprehensively explore the development trends of nonlinear regional economy. In practical prediction, economic prediction model systems are established by using artificial neural networks to make the prediction more accurate.

Aidana et al. [5] proposed an optimal energy scheduling method to address the dynamic economic dispatch problem through using novel genetic algorithm and the short-term load forecasting. Although the hybrid method shows superior forecast ability, time consumption significantly augments due to searching global solutions. Cheng et al. [6] proposed a reinforcement forecasting framework for intraday economic dispatch. The reinforcement forecasting framework is employed aiming to real-time system feedbacks, therefore, a large amount of real-time data is needed as input objects. Qi [7] uses statistical analysis methods to predict economic development, results of which show that the time consumption is less, however, the prediction error is hard to control. Similarly, the pvar model implemented in [8] is used for economic growth trend prediction. And the method in [9, 10]. These above forecast methods belong to tradition forecast methods.

Unlike the above forecast methods, neural networks are highly favored due to their excellent learning and prediction abilities. Such as the Graph Neural Networks [11] used in traffic, the Neural Network [12] applied in signal process, the Deep Neural Networks [13] utilized in diagnostic technologies, and the Deep Neural Networks [14] applied in medical field etc. Generally, different structures of neural networks are commonly used in different application fields, for instance, Jun et al. [15] utilized the Convolutional Neural Networks as a filter, thus analyzing sparse data. Shi et al. [16] designed the Deep Neural Networks for image classification. And the deep neural networks proposed by [17], which is used for classifying breast cancer histopathological images. Similarly, the deep neural networks being used for dynamic MR imaging [18]. For example, DCell based on neural network architecture was implemented by the [19]. Through introducing boolean logic, the pattern trajectory forecasted by DCell is closed to the real trajectory in the cellular growth. Du et al. [20] proposed the Radial Basis Function (RBF) neural network and the Back Propagation (BP) neural network to forecast economy. The compared results show that the forecast ability of RBF neural network is better than that of BP neural network.

Elman neural network is a typical local regression network (global feed forward local current) [21], which can be seen as a recurrent neural network with local memory units and local feedback connections. The Elman neural network proposed by [22] forecasts the security of network system and provides early warnings for network security. Due to using the global solution to train the Elman neural network, the forecast accuracy relies on the global solution, and training consumption also increases. The [23] utilized the Elman neural network combined with a genetic algorithm to forecast traffic. Although the genetic algorithm optimizes network parameters of the Elman neural network, thus significantly increasing predicted precision, the crossover and mutation operations in the genetic algorithm easily affect the forecast performance. Zhang et al. [24] used the IOIF-Elman neural network to predict air quality, addressing the shortcomings both slow convergence and falling into local minimums. Simliarly, these Elman neural networks implemented in [25–28] unveil excellent forecast performance. To optimize Elman neural networks, usually, other methods or other network structures are fused into them. For instance, Masoud et al. [29] proposed the hybrid network based on convolutional neural networks and Elman neural networks. However, the training for the parameters in the hybrid network is complex. Rajesh [30] employed the Memory Recurrent Elman Neural Network, i.e., recurrent neural network is introduced into the Elman neural network. Obviously, these predicted results confirm that Elman neural network is suitable for the data forecast.

### Motivations

The forecast of regional economy relies on historical data of regional economic development, however, due to complex relationships among the historical data, existing analysis methods can not accurately analyze indicators with high regional economic linearity, and gain advanced economic prediction results, so that it is difficult to explore those critical indicators that objectively and comprehensively reflect economic development. Here, the goals in this work are to achieve economy forecast in Guangdong province, and to provide references for economic decision-making in Guangdong province through exploring those critical indicators impacting the economy. To achieve the goals, we designed an Elman neural network with wavelet function, i.e., namely ENN-W. Using the wavelet function can stimulate the forecast ability of Elman neural network, and also improve the convergence speed of the network. From a perspective of the model, neural networks have excellent forecast ability, therefore, we considered the network structures like neural networks. From the view of a method, we utilized the good perception ability of the wavelet functions on the data. Accordingly, through combining both, the economy in Guangdong province can be forecasted, accurately.

### Contributions

We summarized main contributions of this paper.

1. We proposed an Elman neural network with wavelet function to forecast regional economics. Our model not only achieves advanced results to the forecast of individual economic indicator, but also gains satisfactory results to the prediction for multiple economic indicators. We demonstrate that our model is independently of specific scenarios during economic forecast.
2. Our Elman neural network does not adopt a complex design, although complex structure designs can improve forecast capabilities of neural networks. Instead, we took account into a wavelet function instead of the design of complex network structures. This not only reduces the consumption of training time, but also improves forecast accuracy.
3. Our model does not exhibit exponential training time with increasing data volume, i.e., training time emerges a linear trend as data volume augments. Hence, we demonstrate that our model is suitable for the prediction of large-scale datasets.

This paper is arranged as follows. Section 2 illustrates the proposed scheme and model implementation. Section 3 gives experimental setting and designs, and then Section 4 exhibits the experimental results. In Section 5, we discussed the results. Section 6 draws the conclusion and directs future works.

## 2 Methodology

The accuracy of economic forecasting not only relies on an understanding of the history and current situation, as well as the accuracy of the initial data obtained, certainly, but also on the scientific nature of forecasting methods and advanced economic activities and the familiarity with economic theory knowledge [31]. Here, we studied Elman neural networks and wavelet analysis, then built the Elman wavelet neural network to predict the economy.

### 2.1 Scheme’s overview

**Fig 1:**
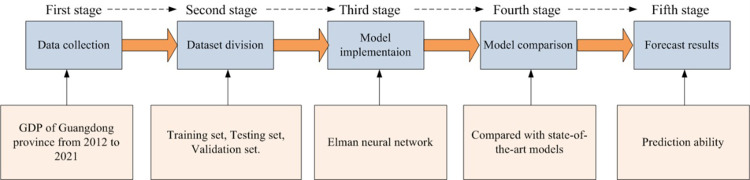
Overview of the proposed scheme.

The proposed overall scheme includes five stages, as shown in Fig 1, where, the data was collected in the first stage. Here, we considered the Gross Domestic Product (GDP) OF Guangdong province from 2012 to 2021. Some incomplete and missed values were filtered, then we performed a normalization operation between zero and 1 on the data.

After processing the collected data, in Second stage, the data was divided into a training set of being used for model training, a testing set of being used for parameter verification, and a validation set of being used to verify the forecasted ability of the model.

Then, in the third stage, namely model implementation, we designed the Elman neural network and chose a wavelet function. There are many types of wavelet functions, from a perspective of the characteristics for wavelet function, and bases on the ascendency of the Elman neural network, Meyer wavelet function was selected.

The four stage is to verify prediction ability of our model, therefore, some state-of-the-art models were used for a comparison. We selected different neural network models being suitable for economic prediction as a comparison. Finally, the forecasted results were displayed and compared in fifth stage.

### 2.2 Model implementation

This section analyzed the structure design of our Elman neural network, the selection of wavelet function, and the training of the model. The details are below.

(1) Model structure

As is known to all, Elman networks have a multi-layer structure like multi-layer forward networks. Based on back propagation network structures, an association layer is added to in the hidden layer as a delay operator to achieve memory function, so that the system can adapt to time-varying characteristics and enhance the global stability of the network. Consequently, it has stronger calculation power than feedforward neural networks, and can solve fast optimization problems.

**Fig 2:**
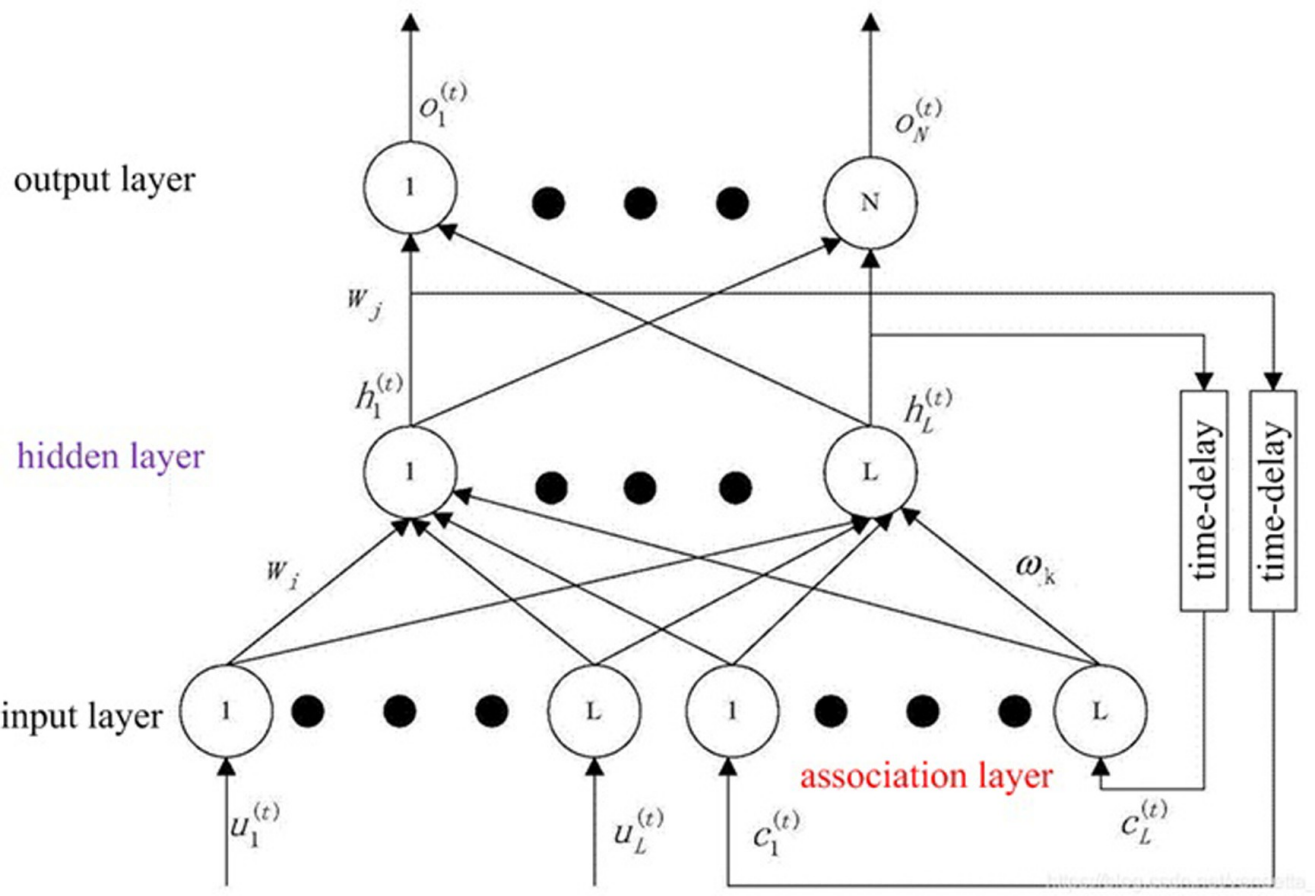
Structure of our network.

Fig 2 displays the structure of our Elman neural network. The main structure is feedforward connection, including input layer, hidden layer, and output layer, connection weights can be learned and corrected. The feedback connection is composed of a set of structural units being used to remember the output value of the previous moment. In addition to the ordinary hidden layer, there is also a special hidden layer, namely the association layer (or connection unit layer), which receives feedback signals from the hidden layer. Each node in hidden layer is connected to a corresponding node in association layer. The correlation layer takes the hidden layer state from the previous time along with the network input from the current time as the input to the hidden layer, i.e., state feedback. The transfer function in hidden layer adopts nonlinear functions, such as Sigmoid function, output layer and association layer adopt linear functions.

We introduced wavelet function into our Elman neural network, thus building the Elman wavelet neural network. The formal description is as follows.


$$o_{j}^{\left(t\right)}=\sum W_{j}^{\left(t-1\right)}h_{j}^{\left(t\right)}$$ (1)

$$H_{j}^{\left(t\right)}=\phi (h_{j}^{\left(t\right)})$$ (2)

$$c_{k}^{\left(t\right)}=H_{k}^{\left(t-1\right)}$$ (3)

$$h_{j}^{\left(t\right)}=\sum {\left[W_{i}^{\left(t\right)}\right]}^{2}*u_{i}^{\left(t-1\right)}+\sum \omega_{k}^{\left(t-1\right)}*H_{k}^{\left(t-1\right)}$$ (4)

Where $W_{j}^{\left(t-1\right)}$ is the connection weight between neurons in hidden layer and those in output layer. $W_{i}^{\left(t\right)}$ is the connection weight between neurons in input layer and those in hidden layer. $\omega_{k}^{\left(t-1\right)}$ is the connection weight between neurons in association layer and those in hidden layer. $u_{k}^{\left(t\right)}$, $h_{j}^{\left(t\right)}$, $o_{j}^{\left(t\right)}$, $c_{k}^{\left(t\right)}$ are input layer, hidden layer, output layer and association layer, respectively. *φ* is wavelet function.

(2) Selection of wavelet function

The selection of wavelet function should be considered from both general principles and specific objects. The general principles are orthogonality, tight support, symmetry, and smoothness. Where orthogonality originates from the simplicity of mathematical analysis and the ease of understanding in engineering applications. Tight support ensures excellent time-frequency local characteristics and are also conducive to algorithm implementation. Symmetry is related to whether the filtering characteristics of wavelets have linear phase, which is closely related to distortion issues. Smoothness is related to the level of frequency resolution. If the smoothness is poor, as transformations increases, the previously smooth input quickly becomes discontinuous, causing distortion during reconstruction. Certainly, it is very difficult to fully meet these characteristics. For example, tight support and smoothness cannot be achieved simultaneously, and tight support of orthogonality makes symmetry impossible. Therefore, only a reasonable compromise solution that can appropriately balance these characteristics can be found. The specific selection should vary depend on the application field, for instance, in terms of image processing, if the purpose is lossless compression, symmetry and smoothness are important.

**Fig 3:**
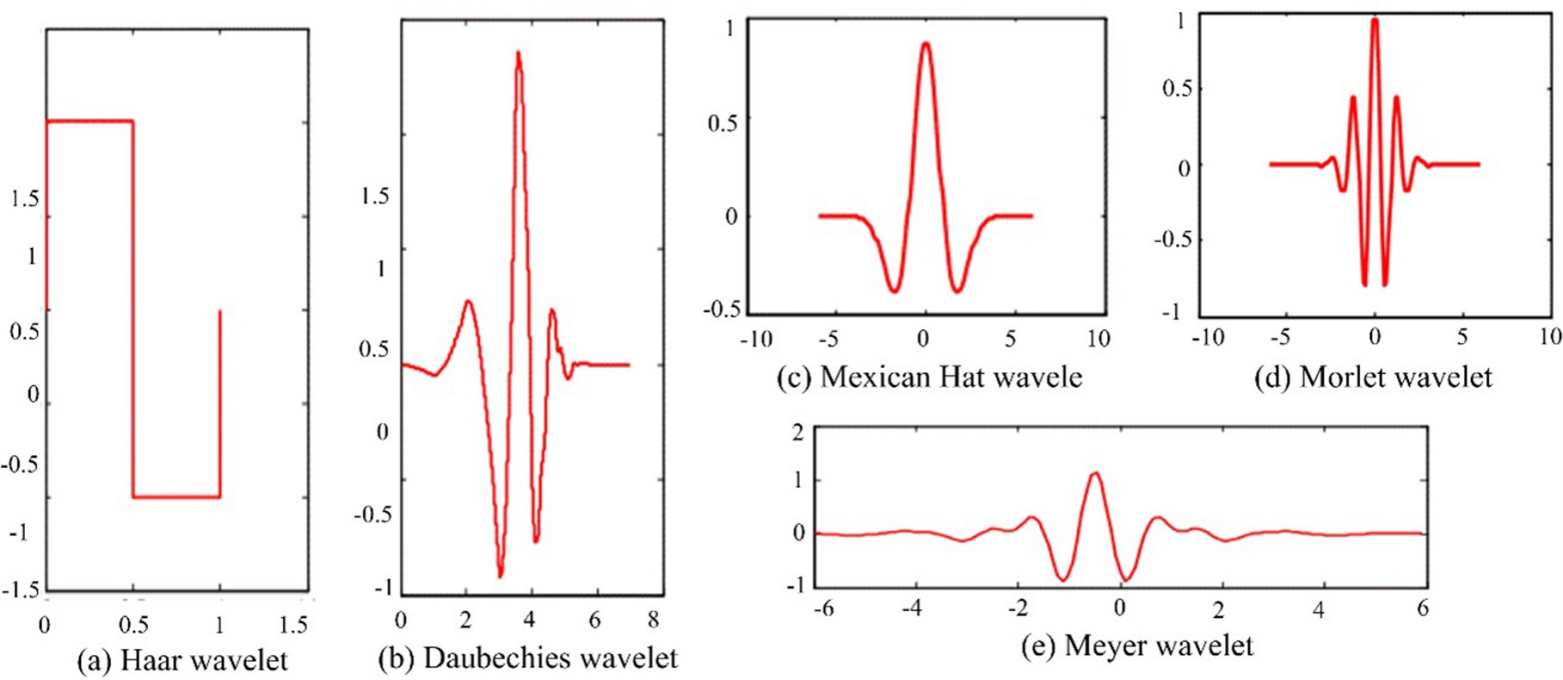
Visualization of five wavelet functions.

Generally, common wavelet basis functions have Haar wavelet, Daubechies (dbN) wavelet, Mexican Hat (mexh) wavelet, Morlet wavelet, Meyer wavelet, etc. Fig 3 unveils the changes of the five wavelet functions on time domains. Haar wavelet function, illustrated in Fig 3(A), is the earliest orthogonal wavelet function with tight support used in wavelet analysis, and it is also the simplest wavelet function. Due to its non-continuity in the time domain, the performance of Haar wavelet function is not very good.

Daubechies (dbN) wavelet, illustrated in Fig 3(B), has good regularity, meaning that the smoothing error introduced by the wavelet as a sparse basis is not easily detected, thus generating relatively smooth in the input reconstruction process. The characteristic of dbN wavelet is that as the order increases, the number of vanishing moments augments. The higher the vanishing moment is, the better the smoothness is. Meanwhile, the stronger the localization ability is in the frequency domain, the better division effect of the frequency band is. However, it weakens the tight support in the time domain, and greatly increases the computational load, and reduces the real-time performance.

As for Mexican Hat wavelet, as shown in Fig 3(C), the characteristic is well localized in both the time and frequency domains. Because there is no scale function, Mexican wavelet function does not have orthogonality.

Morlet wavelet is a single frequency cosine function under Gaussian envelope, as shown in Fig 3(D), it does not have a scaling function and is a non-orthogonal decomposition.

Meyer wavelet, illustrated in Fig 3(E), is not tight support, but the convergence speed is fast. Meyer wavelet function can be infinitely differentiable.

Meyer wavelet can quickly converge, which is beneficial for the training of our Elman network. Moreover, it can be infinitely differentiable, which is helpful for economic forecasting within a certain time (continuity of time). Based on this, we chose Meyer wavelet function. Having that

$$\phi (\alpha )=\left\{\begin{array}{cc} {\left(2\pi \right)}^{\frac{1}{2}}e^{\frac{\alpha }{2}}\sin(\frac{\pi }{2}V(\frac{3}{2\pi }|\alpha |-1)) & \frac{2\pi }{3}\leq \alpha \leq \frac{4\pi }{3}\\ {\left(2\pi \right)}^{\frac{1}{2}}e^{\frac{\alpha }{2}}\cos(\frac{\pi }{2}V(\frac{3}{2\pi }|\alpha |-1)) & \frac{4\pi }{3}\leq \alpha \leq \frac{8\pi }{3}\\ 0 & \left|\alpha |\notin [\frac{2\pi }{3},\frac{8\pi }{3}\right] \end{array}\right.$$ (5)

Where *V* is an auxiliary function to construct Meyer wavelet. As follows

$$V(\beta )=\beta^{4}(35-84\beta +70\beta^{2}-20\beta^{3})\;\beta \in [0,1]$$ (6)

(3) Model training

The training purpose is the determination and correction of network parameters. As follows,

The training process of neural networks training is that of hyper parameters. Some hyper parameters, e.g., the number of neurons, the scale of hidden layers, etc., have a significant impact on prediction ability of neural networks. Our Elman neural network used a single hidden layer, hence, we need to determine the number of neurons and initialization weights.

It is difficult to find universal rules to determine the number of neurons. Generally, the determination of neuron scale relied on pattern characteristics of samples. In view of this, we utilized the following method to determine the number of neurons.

The number of neurons in input layer is calculated by $N_{input}=⟦\sqrt{data\;dimension*data\;volume}⟧$, and sign ⟦ ⟧ represents rounding down. The number of neurons in output layer is calculated by $N_{output}=⟦\sqrt{data\;dimension}⟧$. The number of neurons in hidden layer is determined by $\min(N_{input},N_{output})\leq N_{hidden}\leq 2\max(N_{input},N_{output})$. The number of neurons in association layer is equal to those in hidden layer, i.e., *N*_*ass*_ = *N*_*hidden*_. Initialization weight values is randomly yielded in range between $-\sqrt{N_{input}}$ and $\sqrt{N_{input}}$.

We use the gradient descent method to train the model, and the parameter correction process is as follows.


$$W_{j}^{\left(t\right)}=W_{j}^{\left(t-1\right)}+\Delta W_{j}^{\left(t\right)}$$ (7)

$${\left[W_{i}^{\left(t\right)}\right]}^{2}={\left[W_{i}^{\left(t-1\right)}\right]}^{2}+\Delta {\left[W_{i}^{\left(t\right)}\right]}^{2}$$ (8)

$$\omega_{k}^{\left(t\right)}=\omega_{k}^{\left(t-1\right)}+\Delta \omega_{k}^{\left(t\right)}$$ (9)

$$\Delta W_{j}^{\left(t\right)}=\eta_{1}\Delta W_{j}^{\left(t-1\right)}-\eta_{2}\delta_{W_{j}^{\left(t-1\right)}}$$ (10)

$$\Delta {\left[W_{i}^{\left(t\right)}\right]}^{2}=\eta_{1}\Delta {\left[W_{i}^{\left(t-1\right)}\right]}^{2}-\eta_{2}\delta_{{\left[W_{i}^{\left(t-1\right)}\right]}^{2}}$$ (11)

$$\Delta \omega_{k}^{\left(t\right)}=\eta_{1}\Delta \omega_{k}^{\left(t-1\right)}-\eta_{2}\delta_{\Delta \omega_{k}^{\left(t-1\right)}}$$ (12)

Where *η*_1_, *η*_2_ are learning rate. *δ* in Eqs (10)–(12) is calculated by Eqs (13)–(15). Having that

$$\delta_{W_{j}^{\left(t-1\right)}}={△}_{err}^{\left(t\right)}H_{j}^{\left(t\right)}$$ (13)

$$\delta_{{\left[W_{i}^{\left(t-1\right)}\right]}^{2}}={△}_{err}^{\left(t\right)}W_{j}^{\left(t-1\right)}*\partial \frac{H_{j}^{\left(t\right)}}{\partial h_{j}^{\left(t\right)}}[u_{j}^{\left(t-1\right)}+\sum \omega_{k}^{\left(t-1\right)}*\partial \frac{H_{k}^{\left(t\right)}}{\partial {\left[W_{i}^{\left(t-2\right)}\right]}^{2}}]$$ (14)

$$\delta_{\Delta \omega_{k}^{\left(t-1\right)}}={△}_{err}^{\left(t\right)}W_{j}^{\left(t-1\right)}*\partial \frac{H_{j}^{\left(t\right)}}{\partial h_{j}^{\left(t\right)}}[H_{k}^{\left(t-1\right)}+\sum \omega_{k}^{\left(t-1\right)}*\partial \frac{H_{k}^{\left(t\right)}}{\partial \omega_{k}^{\left(t-2\right)}}]$$ (15)

Where ${△}_{err}^{\left(t\right)}$ is the network error at time *t*. In the process of network training, if the learning rate is too small, the convergence speed of the network is slow. Instead, if the learning rate is too big, the network training might experience oscillations. Hence, learning rates *η*_1_, *η*_2_ are dynamically tuned by Eq (16).


$${\eta_{1,2}}^{\left(t+1\right)}=\left\{\begin{array}{l} (1+\pi ){\eta_{1,2}}^{\left(t\right)}\;{△}_{err}^{\left(t\right)}\geq {△}_{err}^{\left(t+1\right)}\\ (1-\pi ){\eta_{1,2}}^{\left(t\right)}\;{△}_{err}^{\left(t\right)}<{△}_{err}^{\left(t+1\right)} \end{array}\right.$$ (16)

Where *π*∈[1e7,1e5] is a constant.

To accurately forecast the economy in Guangdong province, we selected the GDP of twenty-one cities in Guangdong province from 1993 to 2022. Where the data from 1993 to 2012 was used as a training set, and the data from 2013 to 2017 was used as a testing set. The remaining data, from 2018 to 2022, served as a validation set to validate the predictive ability of our model.

The training algorithm of the model is given in Algorithm 1. The input and output of the algorithm are training set *X* and predicted accuracy, respectively. Hyper parameters are initialized in Step 1, and the division of training set *X* is performed in Step 2 and Step 4. Our model is iteratively trained, as shown in the procedure between Step 5 and Step 11. In the process of the training, using Eqs (7)–(15) to update the gradient. Once the parameters converge, the training is terminated, illustrated in Step 9. We save current trained parameters and obtain current training accuracy, as shown in Step 12 and Step 13. Using testing set to test the trained model, and then testing accuracy is obtained, illustrated in Step 14 and Step 15. Finally, the validation set is used to verify prediction ability of our model, and the prediction accuracy is obtained, illustrated in Step 16 and Step 17.

### Algorithm 1. The training.

Input: training set *X*.

Output: predicted accuracy.

1 Initialization hyper parameters;

2 *X*_*train*_ is obtained by random selecting 70% of *X*; /* training set */

3 *X*_*test*_ is obtained by random selecting 15% of *X*; /* testing set */

4 *X*_*val*_ is obtained by random selecting 15% of *X*; /* verifying set */

5 **for** *i* = 1 **to** *I*_*max*_ **do**:

6 Using training set *X*_*train*_ to train the model;

7 Updating network parameters by Eqs (7)–(15);

8 Obtaining current training accuracy *TrainAcc*(*i*) = *Network*(*X*_*train*_, *i*);

9 **if** *Network*(*X*_*train*_, *i*) converges = = TRUE **then:**

10  **break;**

11 **end for**

12 Save current training parameters;

13 Obtain training accuracy *TrainAcc*(*i*);

14 Use testing set *X*_*test*_ to test *Network*(*X*_*test*_, *i*);

15 Obtain testing accuracy *TestAcc*(*i*);

16 Use validation set *X*_*val*_ to verify *Network*(*X*_*val*_, *i*);

17 Obtain prediction accuracy *PreAcc*(*i*);

## 3. Experimental settings

### 3.1 Datasets

**Fig 4:**
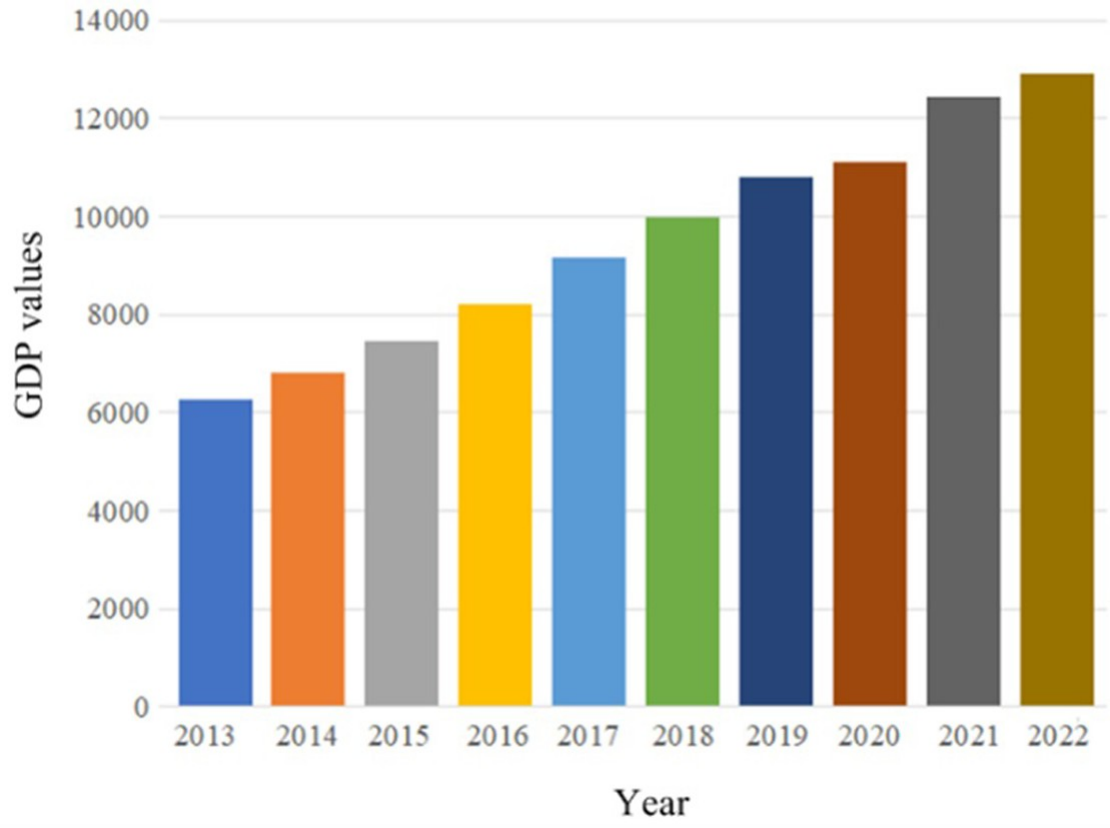
GDP in Guangdong province from 2013 to 2022.

GDP, as the core indicator of national economic accounting, is an important indicator for the measurement of economic status and development level to a country or regions. Regional GDP can serve as a measure of regional economic development. Fig 4 displays the GDP in Guangdong province from 2013 to 2022. In 2022, the GDP reached 12.9 trillion.

To accurately forecast the economy in Guangdong province, we selected the GDP of twenty-one cities in Guangdong province from 1993 to 2022, including Guangzhou City, Shenzheng City, Foshan City etc. Eight indicators regarding the GDP were chose, each of which includes many sub indicators. Table 1 lists the details regarding the GDP for twenty-one cities.

**Table 1:** Dataset details for the GDP in twenty-one cities. There are eight parent indicators, each of which includes many sub indicators.

ID	Cities	Indicators							
1	Guangzhou	Industry	Fixed assets investment	Education, Science, Technology	Agriculture	Culture, Health, Sports	Resources, Environment, Production	Finance, Insurance	Transportation, Tourism, Posts and telecommunications
		Electronic industry, Petrochemical industry, Automotive industry, Advanced manufacturing industry, High-tech manufacturing industry	Railway transportation, Air transportation industry, Water resource management, Environmental governance, Municipal facilities, Urban management, Real estate development	University education, Invention patent	Foodstuff, Fruit, Aquatic product, Meat, Vegetable	Museums, Entertainment facilities, Hospital, Public fitness facilities, Sport Halls	Power supply station, Industrial Park, Sewage treatment plant	Loan, Health insurance, Accident insurance, Property insurance, Other types of insurance,	Expressway, Sea Port, Postal and telecommunications services, Tourist attractions
…	…‥	…‥	…	…‥	…‥	…	…	…‥	…‥
21	Yunfu	…	…‥	…‥	…	…‥	…‥	…‥	…‥

The experimental dataset consists of 36 economic sub-indicators from 21 cities in Guangdong province. We observed the 36 economic sub-indicators within each month as a sub-cycle, and 20 years as a cycle. That is, the data volume is equal to 5040, and the data dimension is equal to 36. Where, the data from 1993 to 2012 was used as a training set, and the data from 2013 to 2017 was used as a testing set. The remaining data, from 2018 to 2022, served as a validation set to validate the predictive ability of our model, illustrated in Table 2. In addition, we performed normalization operations between 0 and 1 on the training set, test set, and validation set.

**Table 2:** Divisions of datasets.

Name	Data	Rate	Role
training set	the data from 1993 to 2012	70%	model training
testing set	the data from 2013 to 2017	15%	parameter testing
validation set	the data from 2018 to 2022	15%	economic prediction

### 3.2 Comparison methods and evaluated indicators

Since our method sufficiently utilized neural network architectures, to a fair and objective comparison, in terms of comparative method, we took account into these competing methods based on neural network architectures. Such as RBF neural network (BP-NN) [14], GA-Elman neural network (GA-ENN) [17], Elman neural network (ENN) [19], and CS-Elman Neural Network (CS-ENN) [21].

Additionally, we use the three metrics Precision, Absolute error, and Relative error as evaluation indicators. Metric Precision is used to evaluate the forecast ability of the five methods (our and the four comparative methods). Using the two metrics Absolute error, Relative error to analyze calculation error of the five methods. The three evaluation metrics can assess the five methods from the two levels, where metric Precision major evaluates the accuracy of regional economic forecast regarding the five methods, while for using the two evaluation metrics Absolute error, Relative error to analyze calculation power of the five methods. Absolute error and Relative error are calculated by Eq (17).


Absoluteerror=|real_value-prediction_value|Relativeerror=|real_value-prediction_value|real_value×100% (17)

### 3.3 Experimental designs

We conducted three groups of experiments to verify the proposed model. As follows

Experiment (I). Economy prediction for Guangdong province. We predicted the GDP in Guangdong province by using the five models (our model and four comparative models), then analyzed the predictive precision of the five models.
Experiment (II). Economy prediction for twenty-one cities. We predicted the GDP in twenty-one cities respectively, then compared the predicted capabilities between the five models.
Experiment (III). Economy indicator prediction. We selected two sub indicators (Sport Halls, University Education) as the predictive objects from the 21 sub indicators, then analyzed the predictive performance of the five models.
Experiment (IV). Analysis for execution efficiency. We observed the execution efficiency of the five models by using the GDP in Guangdong province.

We implemented the five algorithms corresponding to the five methods using Python on TensorFlow framework in Linux Operation System. The experiments were run the same configuration environments. Following that, the five methods were trained by the training set, and then their parameters were verified by the testing set. Once the five methods are well trained, they served as the economic prediction by using the validation set.

## 4 Results and analysis

This section displays the experimental results, indicating that our model defeats the four comparative models for economic predication in Guangdong province, moreover, the executed efficiency also shows the ascendency. The below sub section presents the details.

### 4.1 Economic prediction for Guangdong province

Table 3 unveils the forecasted results regarding the economy in Guangdong province, indicating that our model significantly defeats the four competitors in metric Precision. In term of metric Absolute error and Relative error, the errors obtained by our model is lower that obtained by the four comparative model. Through calculating the GDP values, our model shows excellent predictive ability, especially, in 2021 and 2022, the highest accuracies are obtained, and the minimum Absolute error and Relative error are obtained in 2021 and 2022, respectively. This indicates that our model has ability of economic forecast for Guangdong province.

**Table 3:** Forecasted results of GDP in Guangdong province. The best results are bold and slanted in black. The results are average 10 time.

Year	GDP	Method	Precision	Absolute error	Relative error
2018	9995	ENN-W	***0*.*911***	***18***	***1*.*271%***
		GA-ENN	0.889	36	1.780%
		CS-ENN	0.904	27	1.362%
		GA-ENN	0.901	28	1.669%
		BP-NN	0.890	30	1.722%
2019	10799	ENN-W	***0*.*887***	***33***	***2*.*375%***
		GA-ENN	0.811	66	3.523%
		CS-ENN	0.804	67	5.069%
		GA-ENN	0.879	48	3.919%
		BP-NN	0.806	70	5.871%
2020	11115	ENN-W	***0*.*957***	***11***	***0*.*599%***
		GA-ENN	0.941	16	0.611%
		CS-ENN	0.942	27	0.660%
		GA-ENN	0.934	28	0.686%
		BP-NN	0.935	16	0.620%
2021	12437	ENN-W	***0*.*964***	***7***	***0*.*318%***
		GA-ENN	0.920	22	0.539%
		CS-ENN	0.961	9	0.404%
		GA-ENN	0.946	15	0.505%
		BP-NN	0.952	11	0.411%
2022	12911	ENN-W	***0*.*964***	9	***0*.*233%***
		GA-ENN	0.891	47	0.909%
		CS-ENN	0.903	38	0.882%
		GA-ENN	0.925	29	0.711%
		BP-NN	0.894	59	1.362%

**Fig 5:**
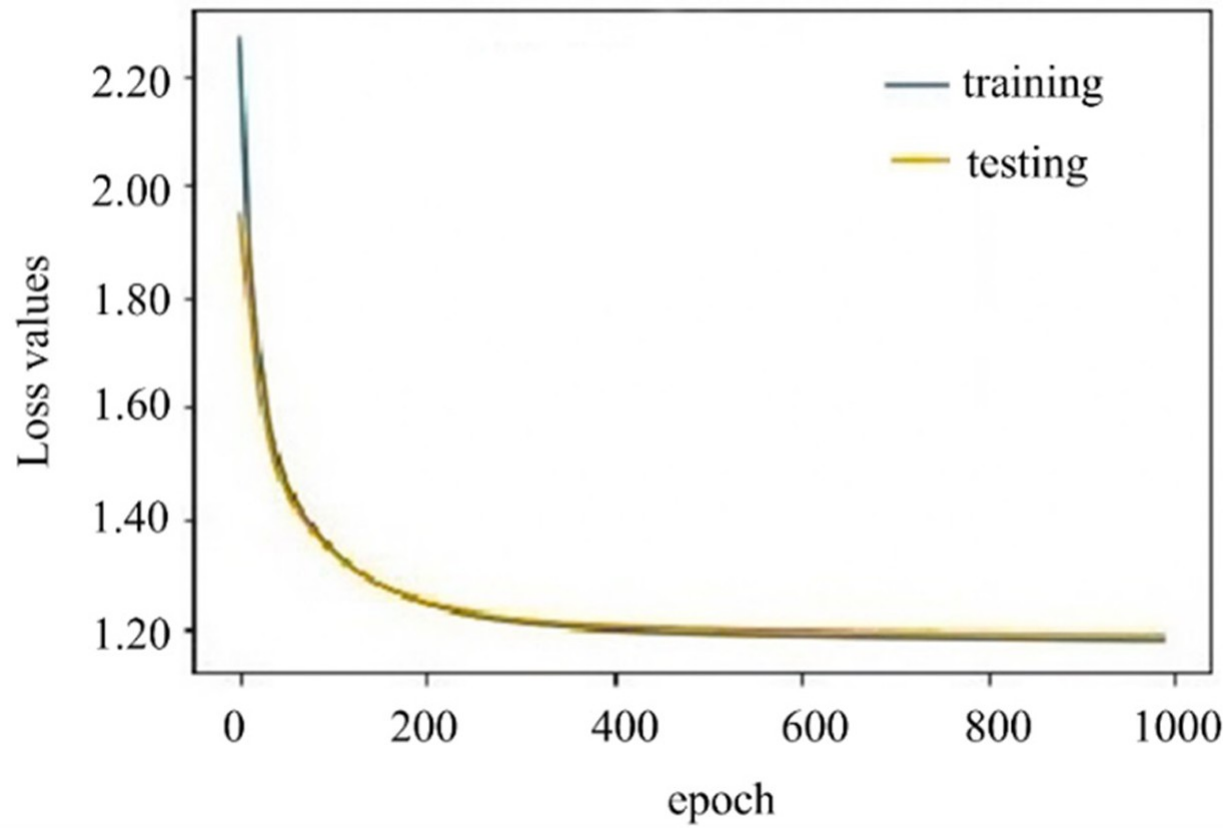
Training and testing loss of the model. Yellow curve line indicates loss values of the testing. Green curve line indicates loss values of the training.

Fig 5 unveils the training loss and testing loss of our model. Our model starts to converge when training epoch reaches 300. Through observing Fig 5, we find that there is no significant oscillation in the loss value curves during model’s training, accordingly, our model does not show over-fitting and is robustness.

### 4.2 Economic prediction for twenty-one cities

**Fig 6:**
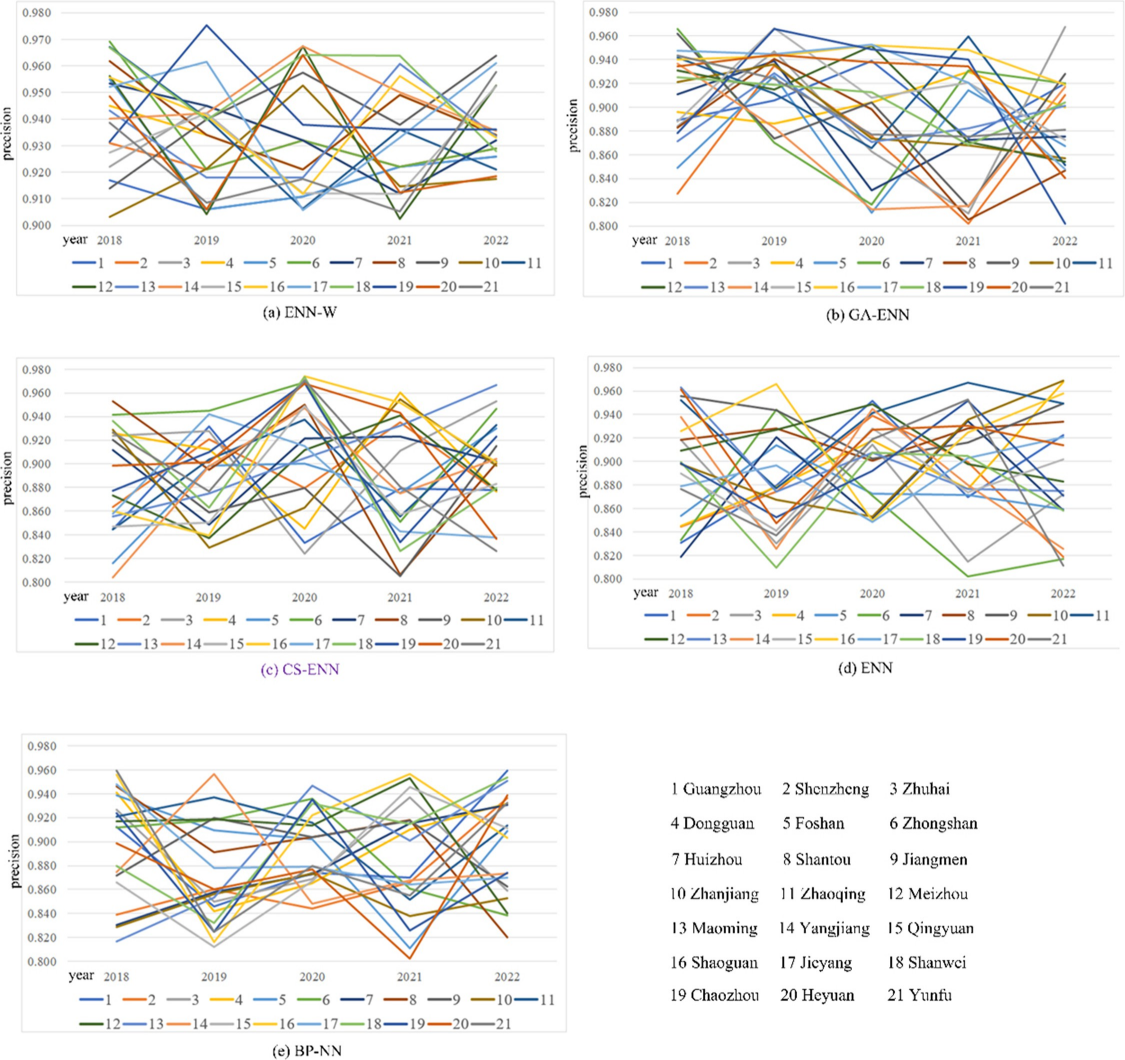
Forecast results for twenty-one cities.

Now, we analyze the forecast result for regional economy. Fig 6 unveils the forecasted results of GDP regarding the twenty-one cities, (here, each city is regarded as a region), results of which show that forecasted accuracies of our model is better than those of the four comparative models in the twenty-one cities. Moreover, our model has a prediction accuracy of over 0.9, of which there is relatively low forecasted accuracy (0.903) in City 10 in 2018, however, there is the highest forecasted accuracy (0.974) in City 19 in 2019. Observing the four competitors, instead, we find that they have a prediction accuracy of over 0.8. From the view of regional economic forecast, our model has excellent ability to forecast urban economy (i.e., economies of different regions). Compared with the economic forecast for Guangdong province, our model has more advantages in predicting urban economy. Since The continuity displayed by the data for each city is better than that displayed by the data for Guangdong province. Meyer wavelet function has a good perception of continuous data.

**Fig 7:**
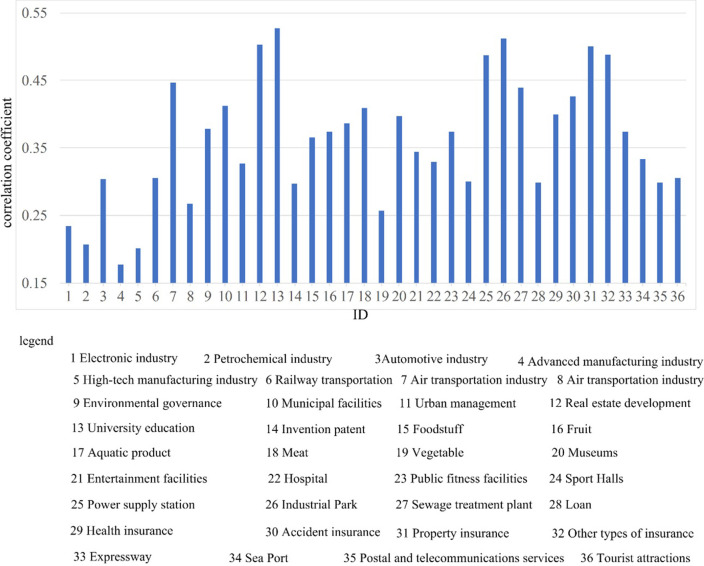
Correlation analysis. The relation of prediction precision and 36 economic sub-indicators.

Additionally, we analyzed the correlation between forecast precisions of our method and the 36 economic sub-indicators, as shown in Fig 7. Through observing the results in Fig 7, we find that the sub-indicator University Education is high related to forecast precisions, i.e., correlation coefficient is equal to 0.527. This means that the investment in education has a major positive impact on regional economic development.

### 4.3 Economic indicator prediction

**Fig 8:**
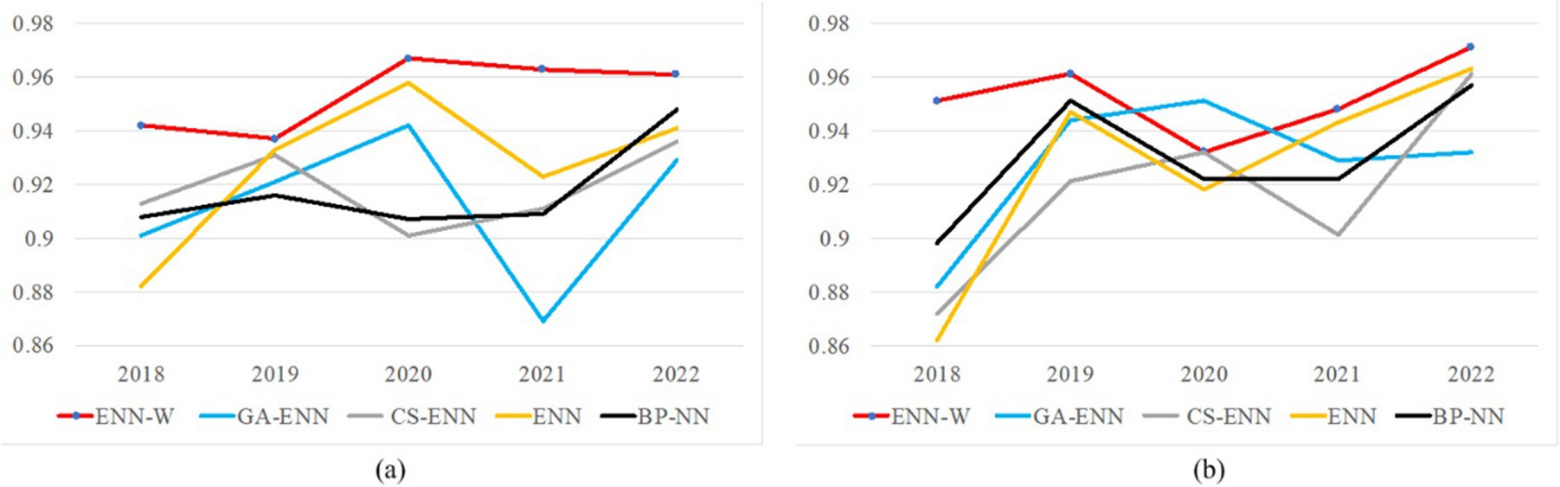
Forecast results of two economic indicators for Guangdong province. (a) is the indicator Sport Halls. (b) is the indicator Universities.

We chose two economic indicators (Sport Halls, University Education for Guangdong province) to forecast, as shown in Fig 8. Forecasted results show that our model defeats the four competitors, and we achieve a prediction accuracy of over 0.9. These confirm that our model achieves good prediction results for both single economic indicators (e.g., Sport Halls, University Education) and multiple economic indicators (e.g., GDP of Guangdong province).

### 4.4 Analysis for execution efficiency

We also explored the executed efficiency of the model, illustrated in Table 4. Whereas, in terms of training consumption, the operational efficiency of our model is not as impressive as its predictive ability. In the process of the training, our model mainly consumes the calculation of wavelet function. The competitor model BP-NN and CS-ENN have the lowest and highest consumption in training, respectively. Nevertheless, these five models have similar consumption in predicting time.

**Table 4:** Running time of the five models.

Models	ENN-W	GA-ENN	ENN	BP-NN	CS-ENN
Training time	387.19s	401.22s	352.38s	289.08s	477.34s
Forecast time	106.77s	111.40s	106.56s	99.48s	122.77s

**Fig 9:**
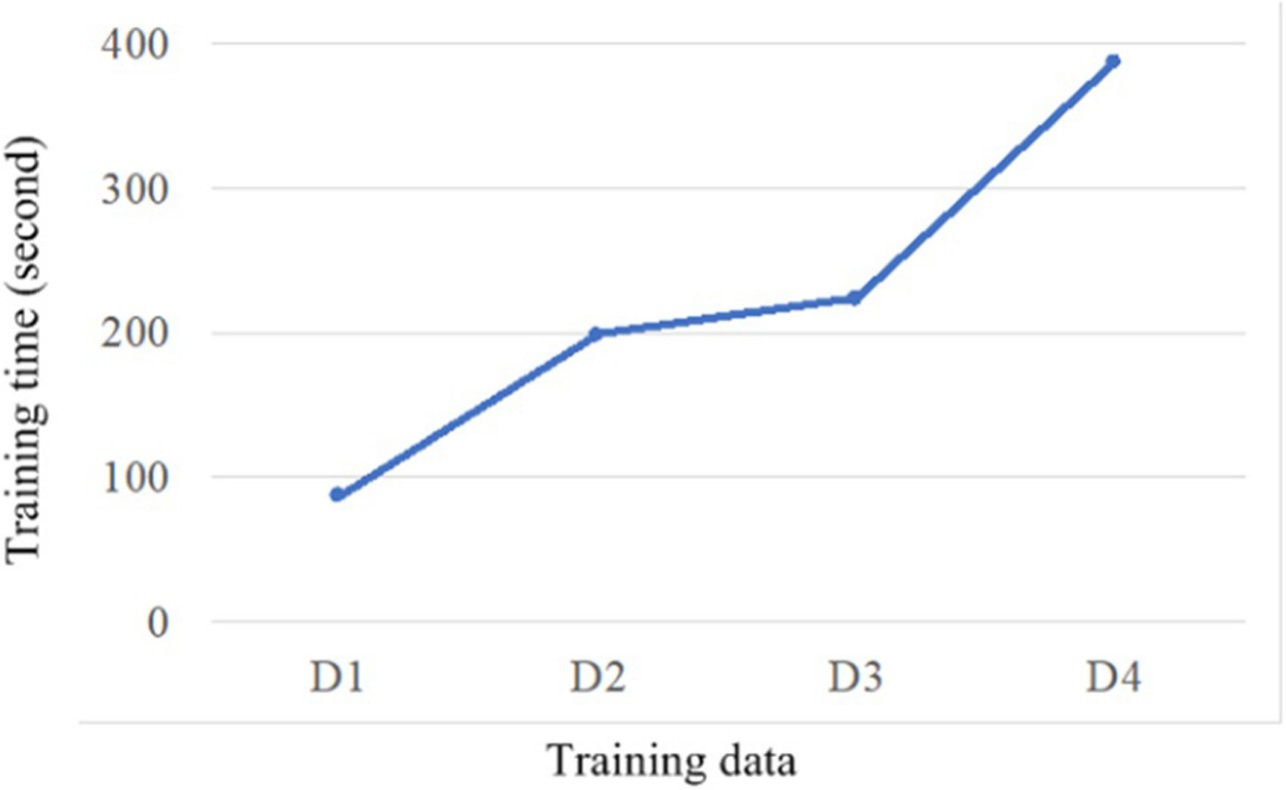
Runing time of our model on the four datasets.

To further explore the training time, our model was trained by using the data D1 (i.e., from 1993 to 1997 for GDP in Guangdong province), the data D2 (i.e., from 1993 to 2002 for GDP in Guangdong province), the data D3 (i.e., from 1993 to 2007 for GDP in Guangdong province), the data D4 (i.e., from 1993 to 2012 for GDP in Guangdong province), respectively. Fig 9 unveils the training time on the four datasets, indicating that our model does not exhibit exponential training time with increasing data volume, i.e., training time emerges a linear trend as data volume augments. Consequently, we demonstrate that our model is suitable for the prediction of large-scale datasets.

## 5. Discussion

### 5.1 Advantages

Compared to the four opponents, the proposed method has ascendency in regional economic forecast in Guangdong province, which can be interpreted as follows.

We utilized Elman neural network as a predictor. Obviously, this structure-based neural networks has natural ascendency in prediction, which is one of the important factors as our predictor. However, the forecast of regional economy is dependent of historical data of regional economic development. To accurately learn complex relations (i.e., those critical representations that objectively and comprehensively reflect economic development) in the historical data, we introduced wavelet function Meyer into Elman neural networks. There are two reasons, of one which is that wavelet function Meyer converges faster than other wavelet functions do, certainly, this is also to allow my model converging quickly. Our final target is forecast regional economy in Guangdong province from 2013 to 2022, clearly, these data between 2013 and 2022 are related to the time, and this is a continuous time cycle. Another ascendency of Meyer wavelet is that it is infinitely differentiable. This ensures that our predictor does not experience significant fluctuations during convergence (i.e., relatively stable, please see Fig 3 regarding the comparison of Meyer wavelet and other wavelets), minimizing our computational errors to the greatest extent possible. From the view of model architectures, the Elman neural network has the characteristics of dynamic operation and has memory function, which is suitable for the forecast of regional economic data. Since economic data belongs to a type of dynamic data being related to time changes, Economic data has continuous characteristics over time cycles. From the perspective of the data, Meyer wavelet exhibits sufficient convergence advantages on continuous data. Based on the above characteristics, we sufficiently combine the advantages of the both, consequently, that is why our method can defeat against the four opponents.

### 5.2 Limitations

Indeed, the proposed method also show limitations. Due to this neural networks-based Elman architectures might fall into local optima, our predictor also faces the risk of encountering local optimum. In the training process of our Elman neural network, gradient descent method was adopted, that is, using Eqs (7)–(15) to update network parameters. This might limit the performance and generalization ability of our predictor. During searching the optimal solution, therefore, our predictor might fall into local minimum. Here, we need to declare that our predictor has a probability of obtaining a local optimal solution, however, this does not mean that our predictor is not ability to obtain a global optimal solution.

We chose 36 economic indicators from 21 cities to form our experimental dataset. The data dimension and data volume of experimental dataset are 36 (i.e., 36 economic indicators) and 5040, respectively, (regarding the detail of dataset, please see Section 3.1), therefore, our experimental dataset is a low-dimensional dataset, but the data volume is sufficiently large. Meyer wavelet can have performance well guarantee in low-dimensional data, however, this well guarantee difficult extends to high-dimensional data due to the curse of dimension. In this work, affected by the characteristic of Meyer wavelet, accordingly, our predictor might not show as well on high-dimensional datasets as on low-dimensional datasets in forecast performance. Additionally, the high dimensionality of the data might also increase the training time of our predictor. In summary, forecast ability and running time of our predictor remains to be further evaluated on high-dimensional datasets.

## 6. Conclusion

This paper proposed an Elman neural network fused with a wavelet function to forecast the regional economy in Guangdong province. To increase the forecast ability of our predictor, we introduced Meyer wavelet function into the Elman neural network. Since Meyer wavelet function not only stimulates the forecast ability of the Elman neural network, but also improves the convergence speed of the Elman neural network. Experimental results show that our model obtains good forecast ability to regional economy, and the forecast accuracy reach 0.971. In terms of economic forecast, our model significantly wins over the competitors. Our model not only achieves advanced results to the forecast of individual economic indicator, but also gains satisfactory results to the prediction for multiple economic indicators. We also find that the investment in education has a major positive impact on regional economic development in Guangdong province, and the both surges positive correlation. As such, our model is independently of specific scenarios during economic forecast. Results also show that our model does not exhibit exponential training time with increasing data volume. Therefore, we demonstrate that our model is suitable for the prediction of large-scale datasets. We also indicate that using wavelet functions can simplify the designed complexity of neural network structures, reducing the training parameter of neural networks.

In future work, we will look at further optimizing the network structures to achieve national economic forecast. To predict more objectively national economy, we will also consider more indicators impacting economic development, such as national policies, and civil consumption capacity, etc. Through extending economic indicators, the data dimension of the experimental dataset can be enlarged. Consequently, we will verify the performance of our predictor on higher dimensional datasets.

## Supporting information

S1 Dataset (XLSX)

## References

[1] Feng Yin, Lishuo Pan, Tianshi Chen, Sergios Theodoridis, Zhi-Quan Tom Luo, Abdelhak M. Zoubir. Linear Multiple Low-Rank Kernel Based Stationary Gaussian Processes Regression for Time Series. IEEE Transactions on Signal Processing, 2020, vol.68, pp.5260–5275.

[2] Huynh Thai Hoc, Radek Silhavy, Prokopova Zdenka, Silhavy Petr. Comparing Multiple Linear Regression, Deep Learning and Multiple Perceptron for Functional Points Estimation. IEEE Access, 2022, vol.10, 112187–112198.

[3] Xinxin Fu, Lu Songhao, Mingzhou Chen, Xuezheng Yue. Optimal Mathematical Modeling of Drug Therapy Based on Gray Correlation Analysis and Neural Network. 2022 IEEE 2nd International Conference on Data Science and Computer Application (ICDSCA), 2022, IEEE, pp.1–8.

[4] Jiang J. Making bureaucracy work: Patronage networks, performance incentives, and economic development in China. American Journal of Political Science, 2018, vol. 62(4), pp. 982–999.

[5] Aidana Kalakova H. S. V. S. Kumar Nunna, Prashant K. Jamwal, Suryanarayana Doolla. A Novel Genetic Algorithm Based Dynamic Economic Dispatch With Short-Term Load Forecasting. IEEE Transactions on Industry Applications, 2021, 57(3), pp. 2972–2982.

[6] Lilin Cheng, Haixiang Zang, Anupam Trivedi, Dipti Srinivasan, Zhinong Wei, Guoqiang Sun. Mitigating the Impact of Photovoltaic Power Ramps on Intraday Economic Dispatch Using Reinforcement Forecasting. IEEE Transactions on Sustainable Energy, 2024, 15(1), pp. 3–12.

[7] Qi Zhao. Research on prediction of enterprise economic growth based on monetary policy regulation. 2020 International Conference on Robots & Intelligent System (ICRIS), IEEE, Sanya, China. 2020, pp. 1–1

[8] Juan Li, ShuFeng Cong. Prediction of financial economic growth trend based on PVAR model. 2021 13th International Conference on Measuring Technology and Mechatronics Automation (ICMTMA), IEEE, Beihai, China, 2021, pp. 1–10.

[9] Zhidong Deng, Nuo Tian, Kunpeng Liu, Di Wu. Trend prediction method of economic fixed base index of power industry based on time series. 2021 International Conference on Wireless Communications and Smart Grid (ICWCSG), IEEE, Hangzhou, China, 2021, pp. 1–4.

[10] Cuiqin Liu. Prediction Method of the Industrial Economic Operation Index Based on an Improved Genetic Algorithm. 2021 IEEE International Conference on Industrial Application of Artificial Intelligence (IAAI), IEEE, Harbin, China, 2021, pp. 1–7.

[11] Ting-Li Huoh, Yan Luo, Peilong Li, Tong Zhang. Flow-Based Encrypted Network Traffic Classification with Graph Neural Networks. IEEE Transactions on Network and Service Management, 2023, vol.20, no.2, pp. 1224–1237.

[12] Shiqi Zhao, Jie Yang, Junzhe Wang, Chaoming Fang, Tengjun Liu, Shaomin Zhang, et al. A 0.99-to-4.38 uJ/class Event-Driven Hybrid Neural Network Processor for Full-Spectrum Neural Signal Analyses. IEEE Transactions on Biomedical Circuits and Systems, 2023, vol.17, no.3, pp.598–609. doi: 10.1109/TBCAS.2023.3268502 37074883

[13] Takafumi Tanaka, Tetsuro Inui, Shingo Kawai, Seiki Kuwabara, Hideki Nishizawa. Monitoring and diagnostic technologies using deep neural networks for predictive optical network maintenance. Journal of Optical Communications and Networking, 2021, vol.13, no.10, pp.13–22.

[14] Yue Deng, Feng Bao, Qionghai Dai, Lani F. Wu & Steven J. Altschuler. Scalable analysis of cell-type composition from single-cell transcriptomics using deep recurrent learning. Nature Methods, 2019, vol.16, no.4, pp.311–314. doi: 10.1038/s41592-019-0353-7 30886411 PMC6774994

[15] Jun Shi, Jianfeng Xu, Kazuyuki Tasaka. SASL: Saliency-Adaptive Sparsity Learning for Neural Network Acceleration. IEEE Transactions on Circuits and Systems for Video Technology, 2021, vol.31, no.5, pp.2008–2019.

[16] Shi Dong, Yifan Sun, Nicolas Bohm Agostini. Spartan: A Sparsity-Adaptive Framework to Accelerate Deep Neural Network Training on GPUs. IEEE Transactions on Parallel and Distributed Systems, 2021, vol. 32, no.10, pp.2448–2463.

[17] Yangqin Feng, Lei Zhang, Juan Mo. Deep Manifold Preserving Autoencoder for Classifying Breast Cancer Histopathological Images. IEEE Transactions on Computational Biology and Bioinformatics, 2020, vol.17, no.1, pp.91–101. doi: 10.1109/TCBB.2018.2858763 30040652

[18] Ziwen Ke, Zhuo-Xu Cui, Wenqi Huang, Jing Cheng, Sen Jia, Leslie Ying, et al. Deep Manifold Learning for Dynamic MR Imaging. IEEE Transactions on Computational Imaging, 2021, vol.7, pp.1314–1327.

[19] Jianzhu Ma, Michael Ku Yu, Samson Fong, Keiichiro Ono, Eric Sage, Barry Demchak, et al. Using deep learning to model the hierarchical structure and function of a cell. Nature Methods, 2018, vo.15, pp.290–298. doi: 10.1038/nmeth.4627 29505029 PMC5882547

[20] Mengru Du. Economic Forecast Model and Development Path Analysis Based on BP and RBF Neural Network. 2023 IEEE 12th International Conference on Communication Systems and Network Technologies (CSNT), 2023, IEEE, pp.619–624.

[21] Elman J L. Finding structure in time. Cognitive Science, vol.14, pp.179–211, 1990.

[22] Yun Kai, Huang Qiang, Ma Yixuan. Construction of Network Security Perception System Using Elman Neural Network. 2021 2nd International Conference on Computer Communication and Network Security (CCNS), 2021, IEEE, 1–8.

[23] Zhilong Zhang, Xianjun Shi, Yufeng Long, Yufeng Qin, Jiapeng Lv, Li Zhao. Network Traffic Prediction Based on Improved GA-Elman Neural Network. 2021 CAA Symposium on Fault Detection, Supervision, and Safety for Technical Processes, 2021, IEEE, pp.1–10.

[24] Yufei Zhang, Jianping Zhao, Honggang Wu, Minghan Gao. A new IOIF Elman neural network for air quality prediction. 2022 2nd International Conference on Consumer Electronics and Computer Engineering (ICCECE), 2022, IEEE, pp.1–12.

[25] Xinlei Cai, Jinzhou Zhu, Jiale Liu, Zijie Meng, Yuhang Huo, Yang Yu. Short- Term Power Prediction Method for Photovoltaic Power Generation Based on Elman Neural Network for Aspen Swarm Optimization. 2023 6th International Conference on Energy, Electrical and Power Engineering (CEEPE), 2023, IEEE, pp.1–10.

[26] Beibei Liu, Diju Gao, Yanyan Sun. A Speed Prediction Method of Electric Propulsion Ship Based on GA-Elman Neural Network. 2022 International Symposium on Sensing and Instrumentation in 5G and IoT Era (ISSI), 2022, IEEE, pp.1–6.

[27] Shuo Li, Huiwen Xia, Li Zhang, Chengwen Dai. Short-term Power Load Forecasting Model Based on CS-Elman Neural Network. 2022 37th Youth Academic Annual Conference of Chinese Association of Automation (YAC), 2022, IEEE, pp.1–7.

[28] Xie Jialing, Shi Weifeng, Bi Zong, Song Tiewei. Research on Marine Electric Load Forecast Based on PSO-Elman Neural Network. 2021 4th International Conference on Energy, Electrical and Power Engineering (CEEPE), 2021, IEEE, pp.1–5.

[29] Masoud Fetanat, Michael Stevens, Pankaj Jain, Christopher Hayward, Erik Meijering, Nigel H. Lovell. Fully Elman Neural Network: A Novel Deep Recurrent Neural Network Optimized by an Improved Harris Hawks Algorithm for Classification of Pulmonary Arterial Wedge Pressure. IEEE Transactions on Biomedical Engineering, 2022, vol.69, no.5, pp.1733–1744. doi: 10.1109/TBME.2021.3129459 34813462

[30] Rajesh Kumar. Memory Recurrent Elman Neural Network-Based Identification of Time-Delayed Nonlinear Dynamical System. IEEE Transactions on Systems, Man, and Cybernetics: Systems, 2023, vol.53, no.2, pp.753–762.

[31] A Editotial. Assessment of Forecast of Social and Economic Development of the Russian Federation for 2019–2024. Finance Theory and Practice, 2019, 23(5):126–130.

